# Real-Time Simulation of a Cerebellar Scaffold Model on Graphics Processing Units

**DOI:** 10.3389/fncel.2021.623552

**Published:** 2021-04-07

**Authors:** Rin Kuriyama, Claudia Casellato, Egidio D'Angelo, Tadashi Yamazaki

**Affiliations:** ^1^Graduate School of Informatics and Engineering, The University of Electro-Communications, Tokyo, Japan; ^2^Neurophysiology Unit, Neurocomputational Laboratory, Department of Brain and Behavioral Sciences, University of Pavia, Pavia, Italy; ^3^IRCCS Mondino Foundation, Pavia, Italy

**Keywords:** cerebellum, spiking network, graphics processing unit, real-time simulation, scaffolding approach

## Abstract

Large-scale simulation of detailed computational models of neuronal microcircuits plays a prominent role in reproducing and predicting the dynamics of the microcircuits. To reconstruct a microcircuit, one must choose neuron and synapse models, placements, connectivity, and numerical simulation methods according to anatomical and physiological constraints. For reconstruction and refinement, it is useful to be able to replace one module easily while leaving the others as they are. One way to achieve this is via a scaffolding approach, in which a simulation code is built on independent modules for placements, connections, and network simulations. Owing to the modularity of functions, this approach enables researchers to improve the performance of the entire simulation by simply replacing a problematic module with an improved one. Casali et al. (2019) developed a spiking network model of the cerebellar microcircuit using this approach, and while it reproduces electrophysiological properties of cerebellar neurons, it takes too much computational time. Here, we followed this scaffolding approach and replaced the simulation module with an accelerated version on graphics processing units (GPUs). Our cerebellar scaffold model ran roughly 100 times faster than the original version. In fact, our model is able to run faster than real time, with good weak and strong scaling properties. To demonstrate an application of real-time simulation, we implemented synaptic plasticity mechanisms at parallel fiber–Purkinje cell synapses, and carried out simulation of behavioral experiments known as gain adaptation of optokinetic response. We confirmed that the computer simulation reproduced experimental findings while being completed in real time. Actually, a computer simulation for 2 s of the biological time completed within 750 ms. These results suggest that the scaffolding approach is a promising concept for gradual development and refactoring of simulation codes for large-scale elaborate microcircuits. Moreover, a real-time version of the cerebellar scaffold model, which is enabled by parallel computing technology owing to GPUs, may be useful for large-scale simulations and engineering applications that require real-time signal processing and motor control.

## 1. Introduction

Flexibility and efficiency are important factors in large-scale computer simulation of spiking neural networks (Brette et al., 2008; Eppler et al., 2009). Flexibility means that spiking neural networks can be built and updated easily and quickly. To ensure flexibility, the simulation software should comprise a number of functional modules that are independent or only loosely dependent, which in turn introduces redundancy that can reduce efficiency in terms of greater memory usage and slower computation. On the other hand, efficiency means that simulation of spiking neural networks can be carried out efficiently in terms of memory and network usage, and computational speed. For example, faster simulation allows us to study biophysical processes that take a long time for hours and days in a reasonable time. A milestone of faster simulation may be real-time simulation. Real-time simulation enables simulated models to be used for engineering applications that require realtime signal processing and motor control, including robot control (Yamazaki et al., in press). To ensure efficiency, simulation software should be written carefully while integrating everything in the code tightly to remove redundancy as much as possible, which in turn could make the code unreadable and difficult for refactoring. Thus, flexibility and efficiency are somewhat conflicting concepts in simulations. How can we compromise between these two factors?

A solution is to use dedicated simulators such as NEST (Gewaltig and Diesmann, 2007), and NEURON (Carnevale and Hines, 2006). These simulators provide integrated environments consisting of easy-to-use interfaces for flexible modeling and also optimized numerical codes for efficient simulation. In fact, these simulators have been used for various projects such as the Human Brain Project (Amunts et al., 2016) in the EU, and Brain Initiatives (Ramos et al., 2019) in the US. Nevertheless, due to large-scale collaborative development, the development of such simulators would not be fast enough to support the latest technologies in the field of high-performance computing (HPC). For example, graphics processing units (GPUs) are becoming popular as hardware for parallel computing, but the NEST simulator has not yet supported it. The NEURON simulator supports GPUs, but the developers had to first extract a module for numerical simulation (Kumbhar et al., 2019), suggesting that they took a modular approach. Therefore, an integrated approach would make the use of the latest HPC technology difficult.

In an intermediate approach known as “scaffolding” (Casali et al., 2019), simulation software is divided into a number of loosely connected or functionally independent modules for neuron and synapse models, connectivity, and numerical methods. An advantage of this approach is that any module can be replaced easily for better descriptions and performance without affecting the other modules. In fact, Casali et al. (2019) replaced simulation modules from/to PyNEST and NEURON with Python interface, and while obtained the same simulation results. Thus, the scaffolding approach is another solution to finding a compromise between flexibility and efficiency.

The present study aims to provide one more proof of concept for the scaffolding approach. In this study, we developed a simulation module that uses GPUs from scratch, replacing a cerebellar scaffold model built in a previous study (Casali et al., 2019), while reusing the other modules as they are. By replacing the simulation module only, we were able to obtain the qualitatively similar simulation results, but the simulations were accelerated by about 100 times, which resulted in faster-than-real-time simulation. Furthermore, we implemented synaptic plasticity, and conducted simulation of a behavioral experiment on eye movement reflex. These results suggest that the scaffolding approach is a promising method for large-scale spiking network simulation.

## 2. Materials and Methods

### 2.1. Overview of the Cerebellar Scaffold Model

**Figure 1:**
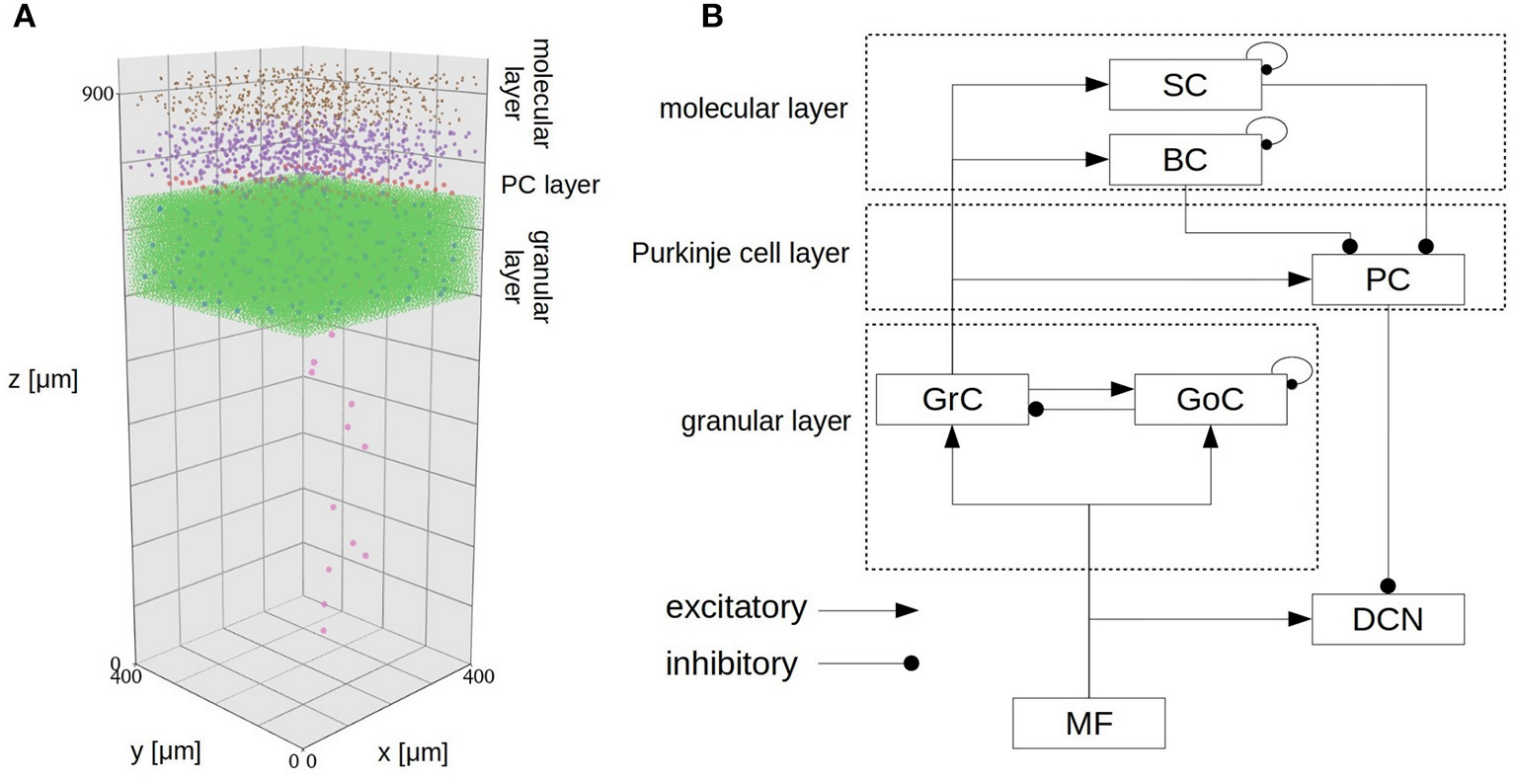
Spatial arrangement of neurons in the cerebellar scaffold model. **(A)** Cell placement in a 3D volume of 400 × 400 × 900 μm^3^. Dots represent neurons, where the colors represent cell types: MF terminal (orange), GoC (blue), GrC (green), PC (red), SC (purple), BC (brown), and DCN (pink). The entire volume contains 96,737 neurons and 4,220,752 synapses. We transposed the z axis to the vertical direction for consistency with the standard atlas. The positions were given by the cell placement module of the original scaffold model (Casali et al., 2019). **(B)** Schematic of connectivity in the same model. Abbreviations are as in the text.

The cerebellar scaffold model (Casali et al., 2019) is a spiking network model of the cerebellar microcircuit built based on the scaffolding approach. According to the approach, the model consists of three modules: a cell placement module, a connectivity module, and a functional simulation module. These modules model and place neurons in a 3D space, create structural connections between pairs of neurons, and simulate dynamics of the network, respectively. The first two are written in Python, whereas the simulation module is written in PyNEST and NEURON. Owing to the modular approach, one can choose appropriate simulator. In fact, one will be able to even replace a cell placement module to use multi-compartment neuron models. The entire circuit includes 7,070 mossy fibers (MFs), 219 Golgi cells (GoCs), 88,158 granule cells (GrCs), 69 Purkinje cells (PCs), 603 stellate cells (SCs), 603 basket cells (BCs), and 12 deep cerebellar nuclei (DCNs). GrCs extend ascending axon (AA) and parallel fibers (PF). All cells are placed in a three-dimensional volume of 400 × 400 × 900 μm^3^ (Figure 1A). The neurons are connected based on known anatomy of the cerebellar circuit (Figure 1B). The original model assumed that glomeruli provide excitatory inputs to DCN, GrC, and Goc, whereas in the present study, we replaced those glomeruli with MFs based on the known anatomy of the cerebellar circuit (Eccles et al., 1967; Ito, 1984).

MFs are modeled as Poisson spike generators. All neurons are modeled as conductance-based leaky integrate-and-fire (LIF) units, and synapses are modeled as conductance-based exponential decay synapses.

A LIF model is defined as follows:

(1) $$\begin{array}{l} C_{\text{m}}\frac{\text{d}u_{i}(t)}{\text{d}t}=-g_{\text{L}}(u_{i}(t)-E_{\text{L}})+I_{\text{e}}+I_{\text{syn}}(u_{i}(t),t)\\ t_{i}^{\left(f\right)}:u_{i}\left(t_{i}^{\left(f\right)}\right)=V_{\text{th}}\\ \underset{t_{i}^{\left(f\right)}\to t,t>t_{i}^{\left(f\right)}}{\lim}u_{i}(t)=V_{\text{r}} \end{array}$$

where *C*_m_, *u*_*i*_, *t*, *g*_L_, *E*_L_, *I*_e_, *I*_syn_, $t_{i}^{\left(f\right)}$, *V*_th_, and *V*_r_ are the membrane capacitance, membrane potential of neuron *i*, time, leak conductance, resting potential, endogenous current, synaptic current, time when neuron *i* fires the *f*th spike, threshold potential, and reset potential, respectively. The first equation describes how the membrane potential is updated. The second equation defines the firing time $t_{i}^{\left(f\right)}$ when the membrane potential reaches the threshold *V*_th_. The third equation resets the membrane potential to the reset potential *V*_r_ after emitting a spike. These values are set for each neuron type (Table 1). A synaptic current and an exponential decay synapse are defined as follows:

(2) $$\begin{array}{r} I_{\text{syn}}\left(u_{i}\left(t\right),t\right)=-\underset{x}{\sum }g_{x}\left(t\right)\left(u_{i}\left(t\right)-E_{x}\right)\\ \;\tau_{x}\frac{\text{d}g_{x}\left(t\right)}{\text{d}t}=-g_{x}\left(t\right)+\underset{f}{\sum }\underset{j}{\sum }w_{ij}\delta \left(t-t_{j}^{\left(f\right)}-t_{\text{dela}{\text{y}}_{ij}}\right) \end{array}$$

where *x* is a synapse label representing either inhibitory (inh) or excitatory (exc), *g*_*x*_, *E*_*x*_, τ_*x*_, *w*_*ij*_, and *t*_delay_*ij*__, are the synapse conductance, reversal potential, decay time constant, synaptic weight between the presynaptic neuron *j* and the postsynaptic neuron *i*, and synaptic delay, respectively. Parameters are set as in Table 2.

**Table 1:** Neuron-specific parameters.

**Type**	**GrC**	**GoC**	**BC**	**SC**	**PC**	**DCN**
N.cells	88,158	219	603	603	69	12
*C*_*m*_ [pF]	3	76	14.6	14.6	620	89
*g*_L_ [ms]	1.5	3.6	1.0	1.0	7.0	1.56
*E*_*L*_ [mV]	−74	−65	−68	−68	−62	−59
Δ*t*_ref_ [ms]	1.5	2	1.6	1.6	0.8	3.7
*I*_*e*_ [pA]	0	36.8	15.6	15.6	600	55.8
*V*_r_ [mV]	−84	−75	−78	−78	−72	−69
*V*_th_ [mV]	−42	−55	−53	−53	−47	−48
τ_exc_ [ms]	0.5	0.5	0.64	0.64	0.5	7.1
τ_inh_ [ms]	10	15	2	2	1.6	13.6

*Abbreviations are as in the text*.

**Table 2:** Synaptic parameters for each connection type.

**Connection type (exc/inh)**	**Weight [nS]**	**Delay [ms]**
MF-GrC (exc)	9.0	4.0
MF-GoC (exc)	2.0	4.0
GoC-GrC (inh)	5.0	2.0
GoC-GoC (inh)	8.0	1.0
AA-GoC (exc)	20.0	2.0
PF-GoC (exc)	0.4	5.0
SC-SC (inh)	2.0	1.0
BC-BC (inh)	2.5	1.0
PF-SC (exc)	0.2	5.0
PF-BC (exc)	0.2	5.0
SC-PC (inh)	8.5	5.0
BC-PC (inh)	9.0	4.0
AA-PC (exc)	75.0	2.0
PF-PC (exc)	0.02	5.0
PC-DCN (inh)	0.0075	4.0
MF-DCN (exc)	0.006	4.0

*Abbreviations are as in the text*.

In the present study, we reused the cell placement module and connectivity module with the same set of parameters. We replaced only the simulation module as in section 2.3.

### 2.2. Parallel Computing on GPUs

Parallel computing is a way to accelerate numerical calculation by dividing a problem into a number of smaller problems and solving them in parallel. GPUs are hardware specialized for computer graphics, but can be used as parallel computing accelerators. A GPU can issue a number of computing entities called “threads” simultaneously, where these threads execute the same function with different input parameters. To date, GPUs play considerable roles in the HPC field.

In this study, we used 4 NVIDIA Tesla V100 GPUs installed into a DGX Station (NVIDIA, 2020b). Briefly, a V100 GPU has 5,120 computing cores, and 16 GB of high-bandwidth memory. The peak performance is 15.7 TFLOPS in single-precision operations. More detailed information of Tesla V100 GPU architecture is described elsewhere (NVIDIA, 2017).

For GPU programming, NVIDIA provides Compute Unified Device Architecture (CUDA), which is a parallel computing platform and a programming model for GPGPU (General-Purpose computing on GPUs) (NVIDIA, 2020a). Programmers can write a code for GPUs with C/*C* + + and CUDA extensions. In CUDA, CPUs and their memory are called “hosts,” whereas GPUs and their memory are called “devices.” Functions computed on GPUs are called “kernels.” Programmers define kernel functions, which are automatically executed by multiple threads with different input parameters. Moreover, although all threads compute the same kernel in principle, when a feature called “streams” is used, different kernels can be executed simultaneously. More detailed documentation is available elsewhere (NVIDIA, 2020a).

### 2.3. A New Implementation of the Simulation Module on GPUs

**Figure 2:**
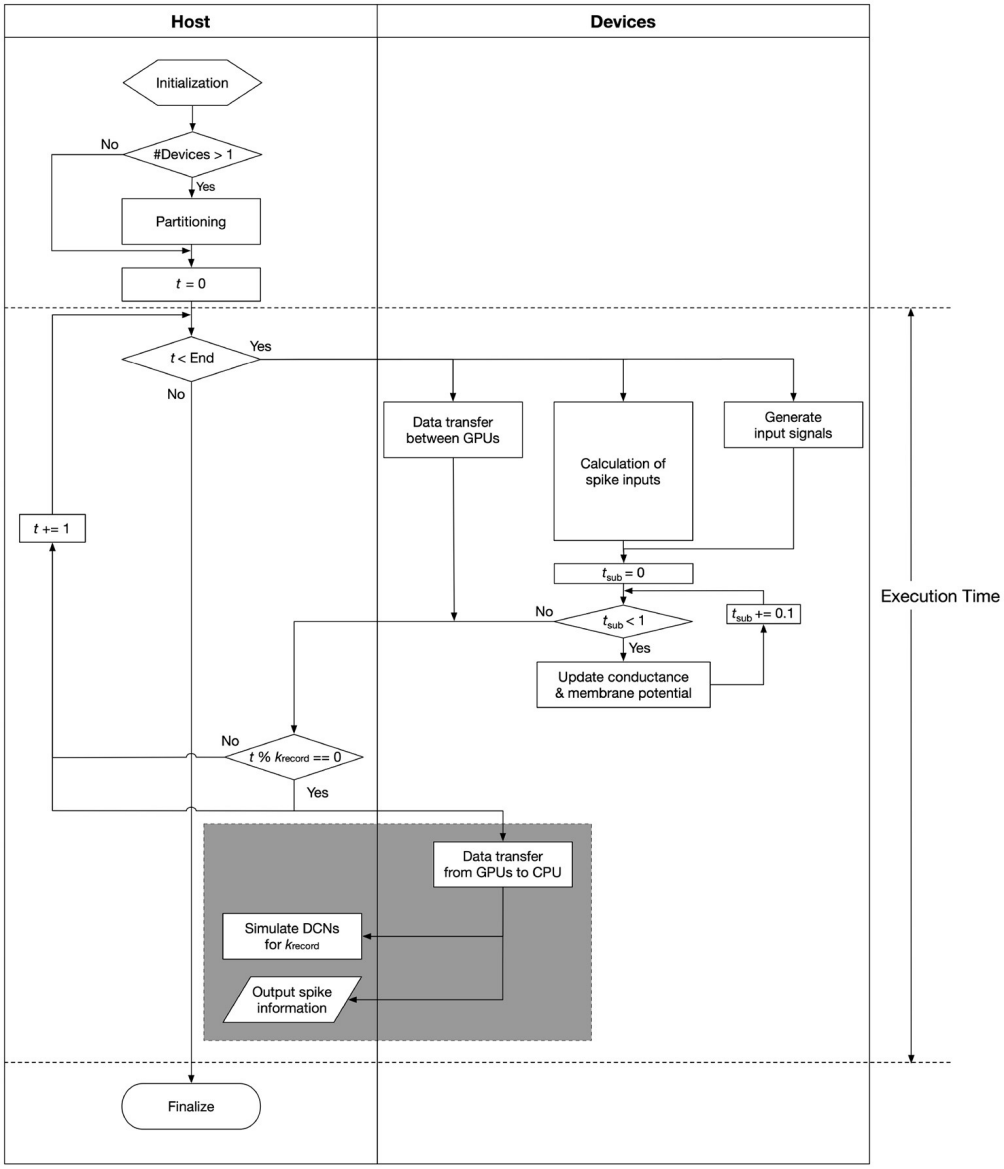
A flowchart of the calculations. Processes in gray rectangle, which manage file I/O and simulation of DCN neurons, are performed in parallel with the rest of the numerical calculations by using pthread library and streams on CUDA. Execution time means the time spent in the area between the dashed lines.

We reimplemented the simulation module of the cerebellar scaffold model, which was written in PyNEST (Eppler et al., 2009), in C language with CUDA extensions from scratch on GPUs. In the new simulation module, Equations (1) and (2) were solved numerically with a forward Euler method with a temporal resolution (Δ*t*) of 0.1 ms, whereas exchanging spike information and performing product-sum operations for synaptic inputs (the 2nd term in the RHS of Equation 2) were made for each 1 ms. These temporal resolutions are identical with the original module. Meanwhile, the original module used the 4th-order Runge-Kutta method instead of Euler method. Euler methods seem to be sufficient for more complicated models such as Izhikevich model (Izhikevich, 2003), so do for LIF models. A flowchart of the calculations is shown in Figure 2.

We used GPUs to perform the above calculation in parallel. Below, we explain the case of a single GPU, and later we will explain how to use multiple GPUs. A basic strategy is to assign 1 neuron to 1 thread and calculate membrane potentials in parallel. During the calculation, the following techniques were used.

First, a connectivity matrix was stored in a compressed sparse row (CSR) format for each pair of presynaptic and postsynaptic neuron types. A CSR format is an efficient way to store sparse matrix. Some other possible formats are coordinates (COO), ELLPACK (ELL), and list of lists (Shahnaz et al., 2005). Second, to generate Poisson spikes on each thread, we used a Philox counter-based random number generator (Manssen et al., 2012). Third, we overlapped spike propagation that requires data transfer between host (CPU) and device (GPU) with calculation of neurons and synapses so as to hide the latency caused by the data transfer. This was achieved by assuming a delay of 1 ms in synaptic transmission (Hines et al., 2011; Igarashi et al., 2019). The overlap of calculation and data transfer was made by using streams in CUDA.

**Figure 3:**
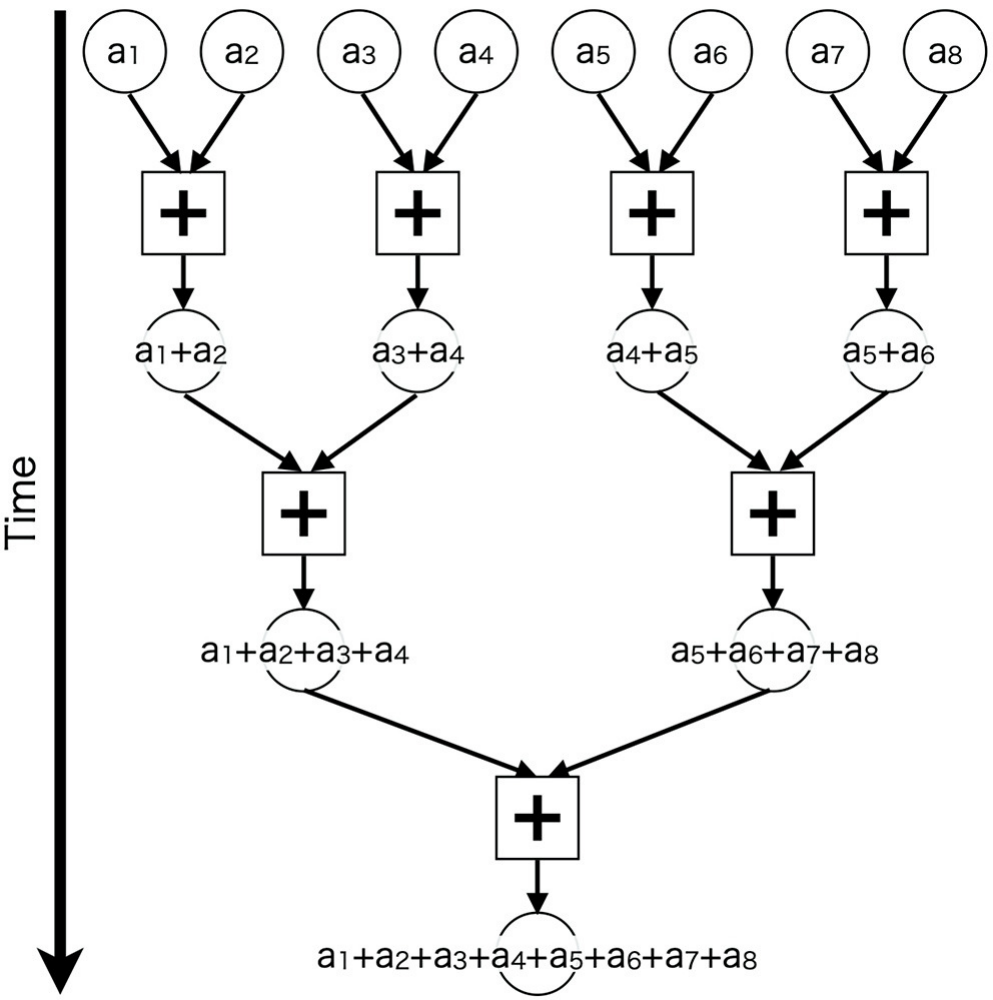
Schematic of parallel reduction. Circles represent values to be added (*a*_1_–*a*_8_) and squares represent add operations (+) assigned to threads. Each thread adds two values in parallel, and the additions are repeated recursively.

Furthermore, we applied parallel computing techniques to calculate synaptic inputs for each neuron. For neurons that are many and have a small number of synapses, such as granule cells, we assigned the calculation for each neuron to each thread. This might be a trivial parallelization. On the other hand, for neurons that are a few and have a large number of synapses, such as Purkinje cells, we used a bunch of threads to calculate synaptic inputs for each cell, and repeated the same calculation to enumerate all cells. Specifically, we used a tree-based approach called “parallel reduction” (Harris, 2011), which reduces product-sum operations recursively (Figure 3). We issued 8,192 threads to perform reduction of 29,196 PF synapses on 1 PC For other neurons, we took an intermediate approach, which was to use a few threads for each cell. More detailed explanation on which parallelization is used for each neuron type is available in Supplementary Material.

In addition to the above GPU-specific techniques, we also used a pthread library in a host code to asynchronously perform data transfer from device to host and file I/O to record spikes of all neurons for each *k*_record_ time step.

To use multiple GPUs, we split an entire network into multiple smaller subnetworks in one dimension along the transversal axis, and allocate each subnetwork to each GPU. Then, we executed the same kernel on all GPUs as in a manner of single instruction, multiple threads (SIMT) over multiple GPUs, although it is possible to execute different kernels on different GPUs. Spikes calculated on each GPU were transferred to its neighboring GPUs for further calculations. We used OpenMP to execute a kernel on multiple GPUs in parallel.

Under multi-GPU setting, DCN neurons must receive inputs from PCs on different GPUs. Because all spike information including spikes emitted by PCs are transferred from GPUs to the host for each 50 ms to generate the output data, we calculated DCN neurons on a host.

### 2.4. Reproducibility of Simulation Results

To examine whether our simulation code reproduces the same results with sufficient accuracy, we conducted the same simulation as in Casali et al. (2019). Briefly, we fed Poisson spikes of 1Hz tonically to all MFs for 1,000 ms as background noise. We also fed phasic Poisson spikes of 150 Hz, starting from 300 ms of the stimulation onset for 50 ms, to MFs at the center of the granular layer within the radius of 140μm. During the simulation, spike trains of all neurons were obtained for these three periods. For each period, we calculated the mean firing rates of all neuron types and their standard deviations, and compared the values with those obtained by the original model. For averaging, we repeated the stimulation 10 times.

### 2.5. Weak and Strong Scaling Properties

Weak scaling and strong scaling are two important measurements for parallel computing applications. In weak scaling, we measure computational time of a simulation while increasing the size of a model and the number of GPUs simultaneously, where the computational load for a computer node is kept the same. Good weak scaling suggests that the size of a model can be increased arbitrarily as long as enough computational resources are available. On the other hand, in strong scaling, we measure computational time while increasing the number of GPUs with a fixed model size. Good strong scaling suggests that the computer simulation can be accelerated when more computer nodes are available.

To examine the scaling, we first prepared three cerebellar scaffold models with the size of 200 × 800 × 900, 400 × 800 × 900, and 800 × 800 × 900 μm^3^, respectively. For weak scaling, we used 1, 2, and 4 GPUs to simulate the three models, respectively. For strong scaling, we performed simulation of the largest model with 1, 2, and 4 GPUs. In the simulation, spontaneous activity with 1 Hz MF spikes were fed as background noise for 10 s of the biological time. The same simulation was repeated 10 times to calculate the mean of the simulation time.

### 2.6. Real-Time Simulation in a More Realistic Scenario

Furthermore, we carried out simulation in a more realistic scenario, which is gain adaptation of eye movements known as optokinetic response (OKR) (Ito, 1984). OKR is an eye movement reflex by which the eyes rotate to the same direction with the visual world's movement so as to reduce the image slip on the retina. If eye movement is too small against the visual movement, an image slip occurs on the retina. The slip generates an error signal fed to PCs via CFs, which in turn induces long-term depression (LTD) at PF-PC synapses. The LTD leads to larger eye movement gain, and further image slips are prevented. This is called gain adaptation of OKR.

To simulate the gain adaptation OKR, we added an inferior olive (IO) to the cerebellar scaffold model and bidirectional plasticity (LTD and long-term potentiation, LTP) at PF-PC synapses as follows:

(3) $$\begin{array}{rc} w_{\text{P}{\text{C}}_{i},\text{P}{\text{F}}_{j}}\left(t+1\right)= & w_{\text{P}{\text{C}}_{i},\text{P}{\text{F}}_{j}}\left(t\right)+0.001\left(w_{\text{init}}-w_{\text{P}{\text{C}}_{i},\text{P}{\text{F}}_{j}}\left(t\right)\right)\\ \;-0.01w_{\text{P}{\text{C}}_{i},\text{P}{\text{F}}_{j}}\left(t\right)\sum_{\Delta t=0}^{50}\text{CF}\left(t\right)\text{P}{\text{F}}_{j}\left(t-\Delta t\right) \end{array}$$

where PF_*j*_(*t*) and CF(*t*) take 1 if PF_*j*_ or CF elicited a spike at time *t*, and 0 otherwise. The 2nd term on the RHS simulates LTP that occurred by firing of a presynaptic PF only (Sakurai, 1987). The 3rd term simulates LTD by conjunctive activation of a CF and a PF (Ito, 2001), that are active 0–50 ms earlier than the CF activation. The *w*_init_ is a constant value representing the initial synaptic weight and was set at 1.0. A synaptic weight for each pair of a PF and a PC is assigned to a thread and updated by the thread independently with every 1 ms.

In the simulation, we fed Poisson spikes that the firing rate modulates from 0 to 30 Hz sinusoidally in 2 s to MFs and that from 0 to 3 Hz to the IO to simulate the visual world movements and the retinal slip errors according to electrophysiological findings (Nagao, 1988). We repeatedly fed these spikes 300 times, and recorded the changes of the firing rates of PCs and DCN cells. Parameters were adjusted according to our previous OKR adaptation study (Yamazaki and Nagao, 2012), since the parameters were different from Casali et al. (2019). We did not include nucleo-olivery inhibitory connections.

## 3. Results

### 3.1. Reimplementation of the Cerebellar Scaffold Model

We have successfully reimplemented the cerebellar scaffold model. Specifically, we implemented the simulation module of the scaffold model on GPUs, whereas the other modules were left unchanged.

**Figure 4:**
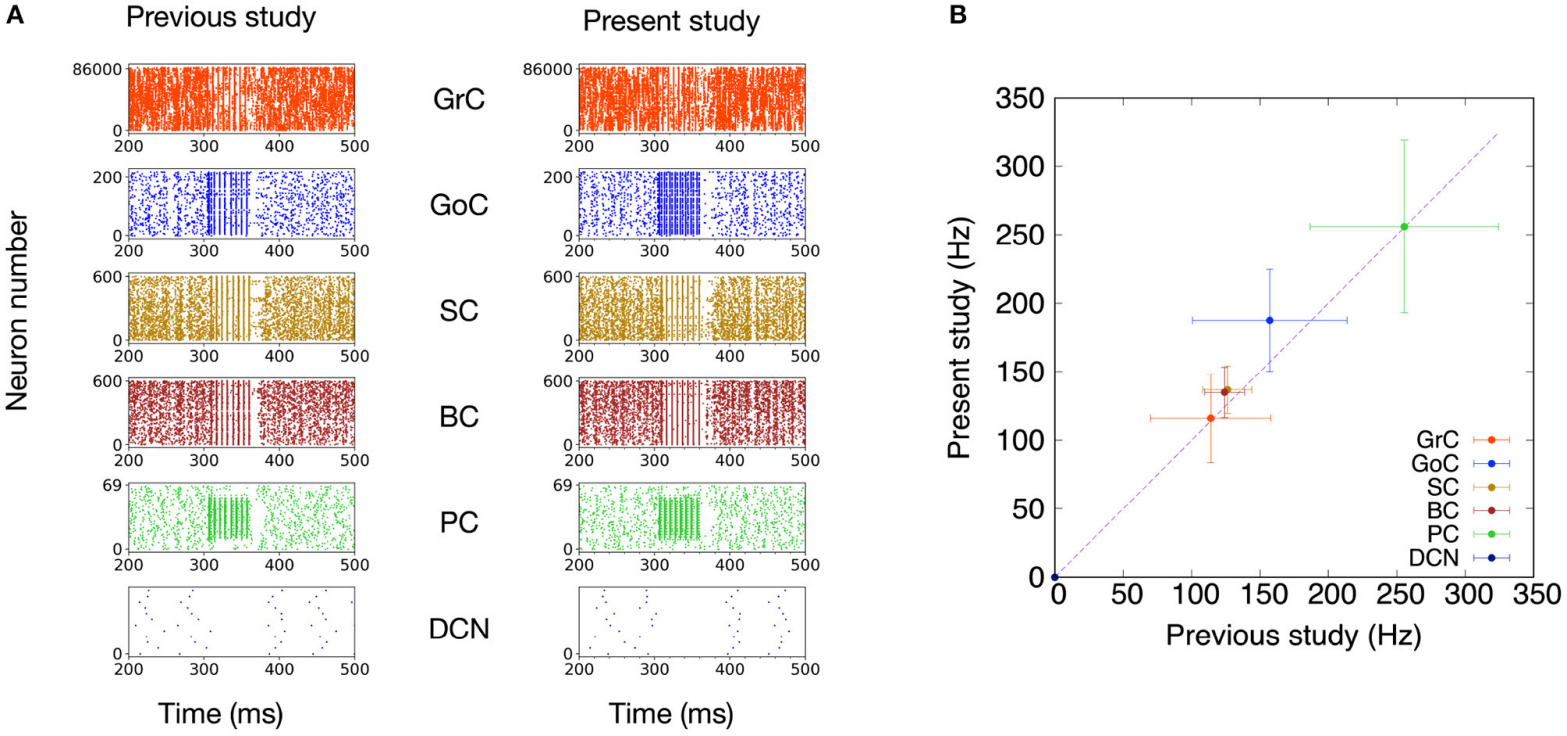
Comparison of simulated neuronal activity between the PyNEST version of the previous study (Casali et al., 2019) and the present study. **(A)** Raster plots of neuron populations in response to MF stimuli (50 ms at 150 Hz on 2,932 MFs) superimposed on 1-Hz background noise. **(B)** Comparison of mean firing rates (Hz) during the input stimulation. The plots compare mean firing rate during the input stimulation for the different neuron populations of the present study with those reported by the previous study (Casali et al., 2019). Dashed line shows identity. Error bars show the standard deviations.

First, we compared the basic dynamics of the previous and present models by presenting the same MF stimuli. The stimulation setting was identical to that in the original paper (Casali et al., 2019) described in section 2.4. Specifically, we plotted spike activities for GrC, GoC, BC, SC, PC, and DCN of the previous (Casali et al., 2019) and present versions, respectively, in response to the MF stimuli (Figure 4A). These raster plots look similar. Then, to quantify the similarity, we calculated the mean firing rates for each neuron type and their standard deviations (Figure 4B). We found that the mean firing rates in one model fall within the range of the standard deviation of the other Here, it should be emphasized that the scaffolding approach intends not to reproduce statistically non-significant results. We will discuss this issue further in section 4. These results suggest that the present model was able to reproduce the network activity of the previous model.

### 3.2. Computational Time

**Figure 5:**
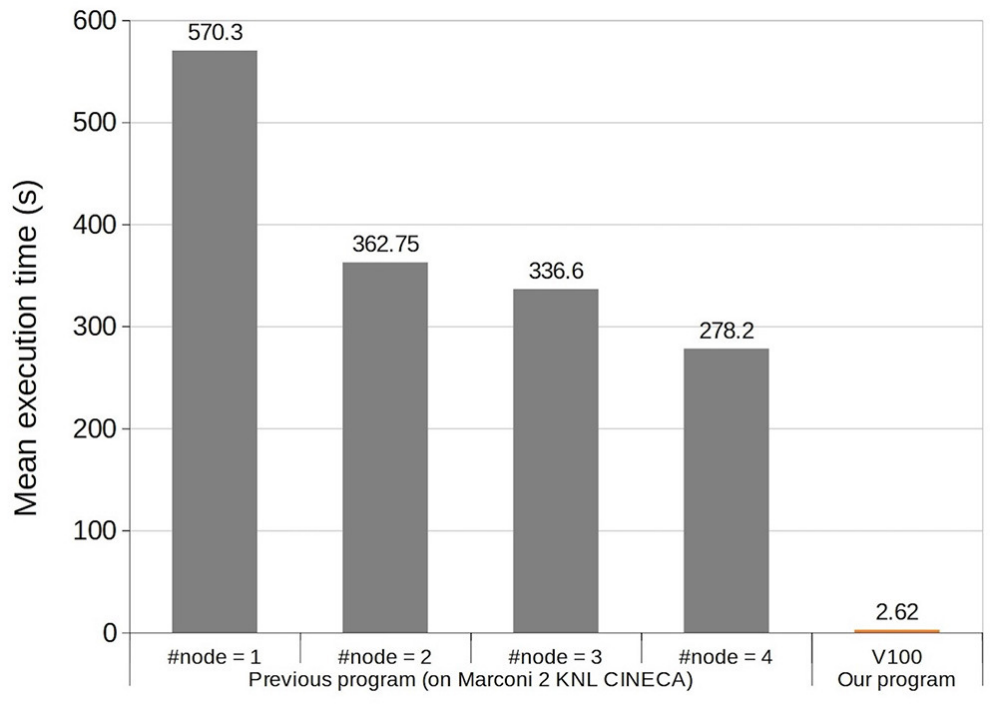
Execution time comparison of 10 s simulation between the previous model (Casali et al., 2019) and ours. The horizontal axis represents four different settings of computers with 1–4 nodes used in the previous study, and our V100 GPU. The vertical axis represents mean simulation time.

Next, we compared computational speeds of the previous and present models. To do so, we fed the same MF stimuli as in the previous section, followed by an additional 9 s of the background noise at 1 Hz, and measured the computational time for the simulation (Figure 5). The previous study reported that a simulation spent 570 s on a single computer node of a supercomputer, which was accelerated to 278 s on 4 nodes on the same computer. On the other hand, our model on a single GPU spent only 2.62 ± 0.15s, resulting in ~217 and 106 times faster speeds, respectively. These results indicate that our model runs four times faster than real time.

### 3.3. Weak and Strong Scaling Properties

**Figure 6:**
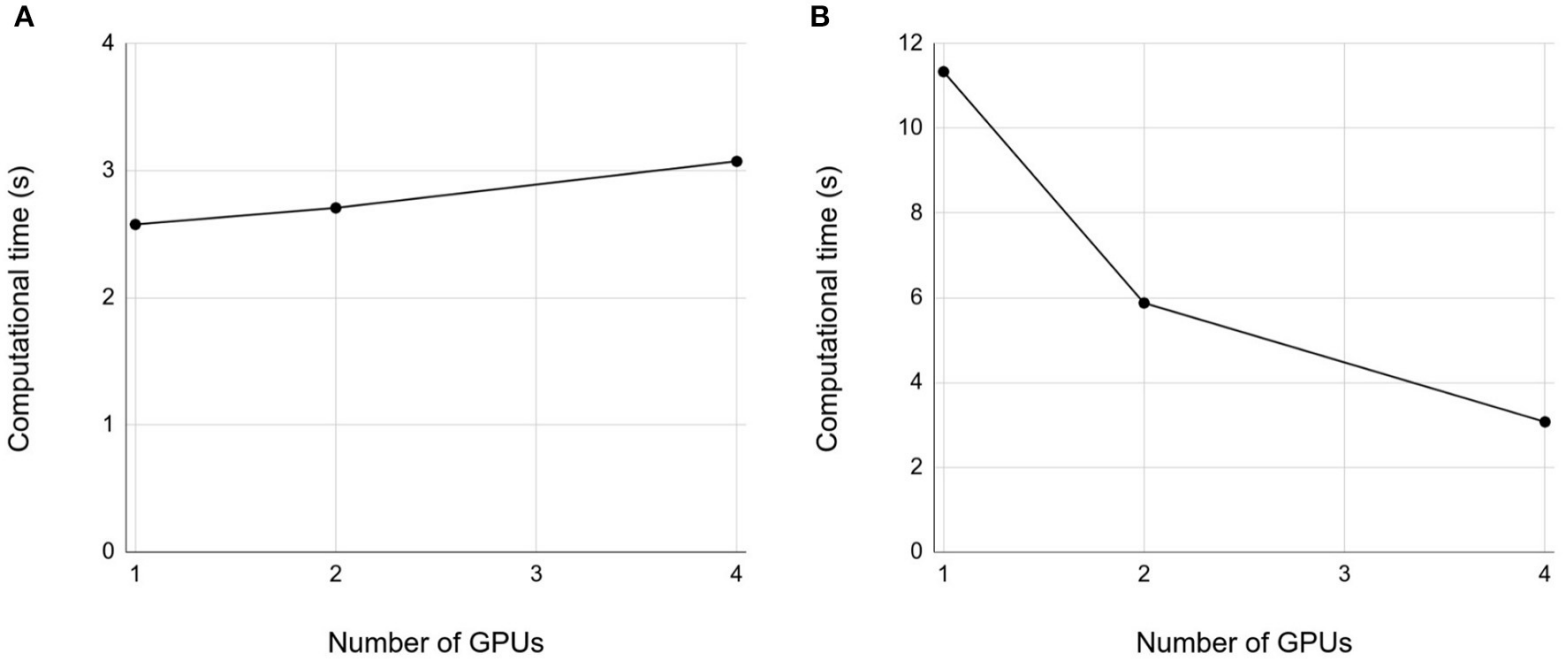
Weak and strong properties. **(A)** Weak scaling. Each GPU simulates the 10 s spontaneous activity of the cerebellar network in 200 × 800 × 900 μm^3^ volume. The horizontal axis is the number of GPUs, and vertical axis is the computational time. **(B)** Strong scaling. A network of the size 800 × 800 μm^3^ volume was decomposed into 1, 2, and 4 subnetworks for 1, 2, and 4 GPUs, respectively. Conventions as in **(A)**.

We then examined weak and strong scaling properties up to 4 GPUs. In both weak and strong scaling, we obtained good scaling properties (Figures 6A,B), respectively. However, the good scaling properties depend on how we split the network into multiple smaller networks to be assigned to different GPUs. We will discuss this issue in section 4.

### 3.4. Faster-Than-Real-Time Simulation of OKR Gain Adaptation

**Figure 7:**
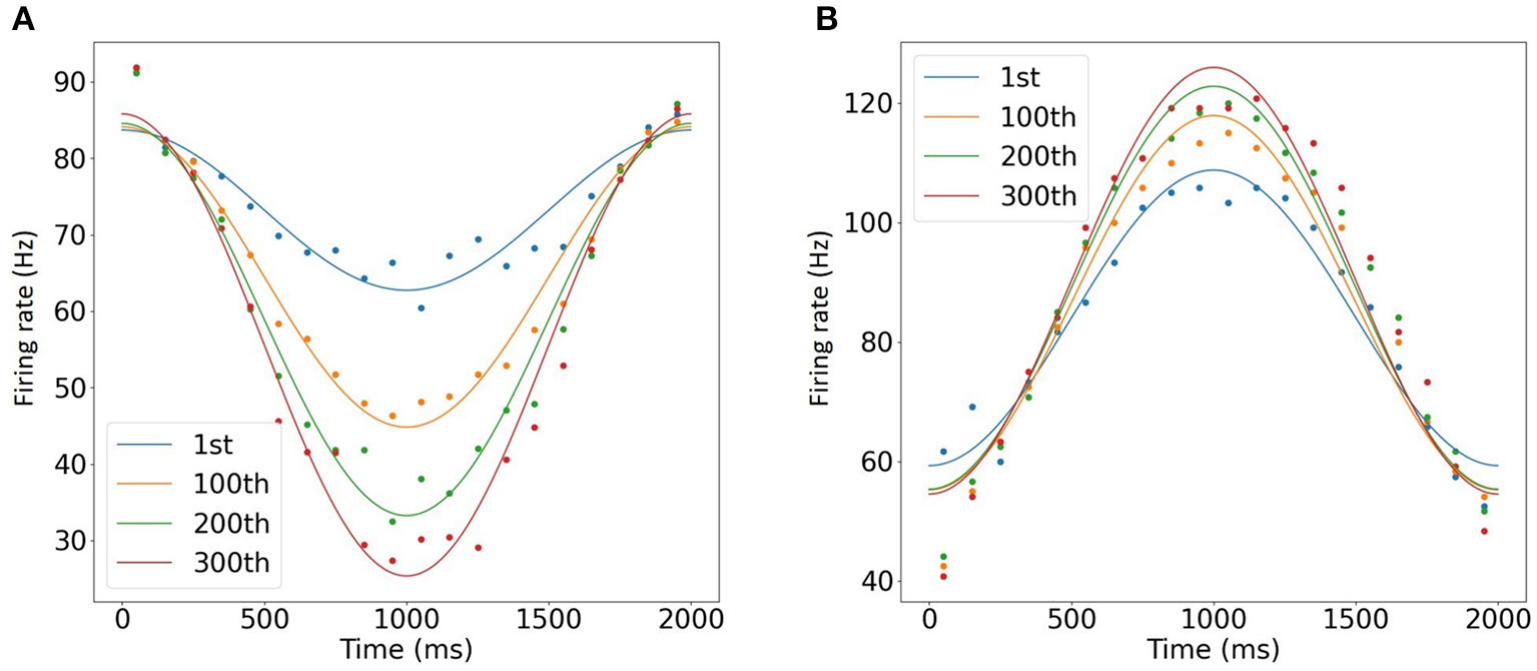
Simulation of OKR adaptation. **(A,B)** Learned change of the mean firing rates of PCs **(A)** and DCNs **(B)** at the 1st, 100th, 200th, and 300th trials of MF signal oscillation (blue, orange, green, and purple, respectively). Dots represent firing rates every 100ms, whereas lines show the data fitted with cosine functions. The horizontal axis represents time for 1 trial, and vertical axis represents firing rate in Hz.

Finally, we conducted computer simulation of OKR gain adaptation. To simulate synaptic plasticity at PF-PC synapses, we added an IO cell. In response to sinusoidal MF signal, PCs exhibited a sinusoidal activity pattern with the opposite phase, whereas DCNs were activated in phase. Furthermore, during 300 trials, PCs decreased the firing rate, where the minimal firing rate changed from 60 to 24 Hz (Figure 7A). On the other hand, DCNs increased the firing rate from 105 to 124 Hz (Figure 7B), which could correspond to gain increase in OKR (Nagao, 1988). Furthermore, a simulation of a 2 s trial was completed within 750 ms, suggesting that faster-than-real-time simulation was achieved.

## 4. Discussion

### 4.1. Proof of Concept of the Scaffolding Approach

The scaffolding approach aims to make neuron models, placements, connectivity, and numerical methods replaceable without affecting the others. Our study is a proof of concept of this approach. In fact, we were able to replace the original simulation module with our version written from scratch that worked efficiently on GPUs. We were able to accelerate simulations faster than real time, while reproducing the same simulation results with the previous study, and further obtain good scaling. Real-time simulation is considered a milestone for simulation speed. Faster than real-time simulation will allow further elaboration of the present model while still keeping real time simulation. These results suggest that the scaffolding approach is useful as a compromise between flexibility of modeling and efficiency of simulation.

### 4.2. Better Decomposition of a Network for Better Scaling

To obtain good parallel computing performance and scaling, it is crucial to consider how to decompose a large network into a set of smaller subnetworks. In neural network simulation, three types of decomposition have been used: random, 2-D, and 1-D. In random decomposition, a network is split into multiple subnetworks that contain almost the same numbers of neurons and synapses for load balancing. This method is usable for any type of neural network, even a network that does not have spatial structures. The NEST simulator has been using this method (Jordan et al., 2018). In 2-D decomposition, a network with spatial cellular and synaptic placements is split into multiple smaller 2-D tiles. This method is useful when a network has spatially regular connections spanning in 2-D or 3-D, and the connections are rather short. The MONET simulator uses this method (Igarashi et al., 2019; Yamaura et al., 2020). In this study, we used 1-D decomposition. We split our network model along the transversal axis. This was efficient for cerebellar networks, because in the cerebellar cortex, PFs run several mm along transversal direction in parallel (Eccles et al., 1967), suggesting that splitting the network in the perpendicular direction (i.e., sagittal direction) result in a large number of communications for spike propagation. However, when we are able to simulate a sufficient size of a network that is larger than a few cm on a GPU, 2-D decomposition will be better than 1-D, because PF spikes do not propagate longer than 1 cm.

### 4.3. Comparison With Other Models

Various computational models of the cerebellar microcircuit have been proposed. Medina et al. (2000) was the first large-scale spiking network model with 10,000 neurons for delay eyeblink conditioning, which was later reimplemented on a single GPU with 1 million neurons (Li et al., 2013). Yamazaki and Tanaka (2007) and Yamazaki and Nagao (2012) built another spiking network model with 100,000 neurons that integrates gain and timing learning mechanisms. These models were also reimplemented on a single GPU (Yamazaki and Igarashi, 2013), multi GPUs (Gosui and Yamazaki, 2016), and a supercomputer with 1,024 PEZY-SC processors (Yamazaki et al., 2019).

All these GPU versions ran in real time as did the present study. The last one was composed of 0.1 billion neurons while retaining real-time simulation. That is roughly the same size as a cat's entire cerebellum. The simulation codes of these models were written by the authors specially from scratch without using general-purpose simulators. Writing special codes enables us to harness the maximal performance of computers while giving up flexibility and reusability of the codes. On the other hand, by using general-purpose simulators, scientists can focus on modeling while ignoring all the other issues which are not related to neuroscience, such as writing programs, debugging, numerical methods, accuracy, and stability. Yamaura et al. (2020) used a general-purpose simulator MONET and built a human-scale network model composed of 68 billion spiking neurons on the K computer, which was the previous Japanese flagship supercomputer, although the computer simulation was about 600 times slower than real time. A drawback of using general-purpose simulators is much lower performance compared with special-purpose programs.

The scaffolding approach, which was originally proposed by Casali et al. (2019) and augmented in the present study, is an intermediate approach. In this approach, simulation codes are decomposed into a few functional modules which are almost independent but loosely connected. Each module can be either written from scratch as in the present study or implemented using existing simulators as in Casali et al. (2019). In fact, our simulation module is reusable for other types of networks that are generated by a connectivity module. A similar approach has been demonstrated by Brian2GeNN (Stimberg et al., 2020), which generates a custom C++ code with GPU support from a script written for Brian simulator (Goodman and Brette, 2008). While the scaffolding approach separates cell placement and connectivity into different modules, Brian integrates them. In this sense, the scaffolding approach might be more flexible. Meanwhile, Brian2GeNN supports only a single GPU at the time of writing. In this way, the scaffolding approach provides a good compromise between flexibility and performance.

Furthermore, the scaffolding approach may allow us to elaborate a model gradually as easily as general simulators, while realizing efficient simulation comparable to custom-code simulators. For example, single-compartment models with more realistic internal parameters have already been integrated in a scaffold model (Geminiani et al., 2019). Moreover, multi-compartment neuron models, such as a PC model (De Schutter and Bower, 1994a,b; Masoli et al., 2015; Masoli and D'Angelo, 2017), a GoC model (Solinas et al., 2007a,b), a GrC model (Diwakar et al., 2009; Dover et al., 2016; Masoli et al., 2020), and IO models (Schweighofer et al., 1999; De Gruijl et al., 2012), will be integrated. Integrating these elaborated models would allow us investigate more detailed network dynamics including synaptic plasticity (Casali et al., 2020) as well as intracellular dynamics simultaneously.

### 4.4. Limitations

Several limitations exist on our simulation module and the scaffolding approach itself.

Our simulation module so far implements only conductance-based LIF model for neurons, conductance-based exponential decay synapses, and Euler methods for numerical integration so as to simulate the cerebellar model implemented in Casali et al. (2019). Such simple implementation helped to achieve real-time simulation. On the contrary, if we implement nonlinear models such as Hodgkin-Huxley type models and more precise numerical methods such as a 4th-order Runge-Kutta method, they may slow down numerical simulation than real time. However, currently 95% of execution time is occupied by product-sum operations for synaptic inputs. Therefore, incorporating more elaborated neuron/synapse models and precise numerical methods will not affect the simulation time significantly. Furthermore, our simulation module includes some model-specific optimizations for the cerebellar model such as parallel reduction, 1-D decomposition, and DCNs' simulation at host. Applying these optimizations to other types of network models would cause a slow down.

On the other hand, when a network model employs randomness in the simulation such as input noise, the scaffolding approach does not guarantee individual spike timing-level reproducibility across different simulation modules, because randomness is managed by individual simulation modules. The scaffolding approach intends not to provide completely identical results across multiple simulation modules, but to only provide statistically non-significant results.

### 4.5. Future Directions

We are now able to simulate in real time a cerebellar circuit with multiple functional modules, at as large a scale as we have a sufficient number of GPUs, owing to the weak-scaling property. The model will be able to run efficiently on supercomputers with multiple GPUs, such as JUWELS (Forschungszentrum Jülich, 2019) and JURECA (Krause and Thörnig, 2016) in Julich supercomputing center, and Germany managed under the Human Brain Project.

Because the model will consist of multiple functional modules, which could learn internal models independently, the model will provide a means to investigate how multiple internal models work together synergistically to perform a complex task, which has been postulated by MOSAIC models (Wolpert and Kawato, 1998; Haruno et al., 2001). Contrary to the modular computing approach, Michikawa et al. (2020) recently reported that an ultra wide-field Ca^2+^ imaging procedure over the entire cerebellar cortex revealed that all microzones are always activated simultaneously, suggesting that all cerebellar modules function in a holistic manner. This study implies that many rather than a small subset of microcomplexes share functions for a given task, but how? To address these questions computationally, our cerebellar model would be a useful tool. Moreover, we could even replace neuron models with more elaborated versions, which would be important for studying how intracellular Ca^2+^ signals spread over the entire cerebellar cortex.

Furthermore, real-time computing capability will also allow us to adopt the cerebellar model as a controller of hardware robots, as in the previous studies (Garrido et al., 2013; Yamazaki and Igarashi, 2013; Naveros et al., 2014; Casellato et al., 2015; Pinzon-Morales and Hirata, 2015; Xu et al., 2018). An advantage of the present model over the previous models is that although the previous models could control only one parameter, because they have only one output channel, the present model can control multiple parameters. This will enable us to control complex robots that have many degrees of freedom. Furthermore, learning capability of the present model would enable such robots to learn appropriate behaviors autonomously. Although traditionally the cerebellum is considered a supervised learning machine that requires an external teacher, a recent study proposes that the cerebellum is a reinforcement learning machine (Yamazaki and Lennon, 2019) that does not require such teachers. The cerebellum as a reinforcement learning machine will be a promising candidate for such autonomous robot learning. Along with the development of spiking neural networks acting as learning machines, development of hardware for emulating such spiking neural networks has been advanced rapidly (Monroe, 2014). Such hardware called “neuromorphic processors” aim to emulate spiking neural networks in real time with ultra low power for mainly edge computing (Furber et al., 2014; Merolla et al., 2014; Friedmann et al., 2017; Davies et al., 2018; DeBole et al., 2019). Machine learning study and computational neuroscience study would be integrated more tightly for real world applications.

## 5. Conclusion

The present cerebellar model is flexible and extendable, owing to the scaffolding approach. It is also efficient, owing to the parallel computing capability of GPUs. The model will provide a means to further investigate the role of the cerebellum in motor or non-motor learning computationally. Finally, the source code is available at the author's GitHub^1^.

## Data Availability Statement

The raw data supporting the conclusions of this article will be made available by the authors, without undue reservation.

## Author Contributions

TY and ED'A conceived and designed the research. CC contributed the source code of the original scaffold model. RK wrote the code, performed simulation, and analyzed the data. RK and TY wrote the original draft. RK, CC, ED'A, and TY discussed on the draft and revised for the final version. All authors contributed to the article and approved the submitted version.

## Conflict of Interest

The authors declare that the research was conducted in the absence of any commercial or financial relationships that could be construed as a potential conflict of interest.

## Footnotes

^1^https://github.com/Rkuriyama/Cerebellar-Scaffold-Model-Simulation-on-GPU/
