# Supplementary material for: Real-Time Simulation of a Cerebellar Scaffold Model on Graphics Processing Units
Source: Front Cell Neurosci. 2021 Apr 7;15:623552. doi: 10.3389/fncel.2021.623552 (PMC8058369; doi:10.3389/fncel.2021.623552)
Supplement: Supplementary file 1 [file Data_Sheet_1.PDF]

Supplementary Material

1 PARALLELIZATION METHODS FOR CALCULATING SYNAPTIC INPUTS

As mentioned in Section 2.3, we chose a parallelization method to calculate synaptic inputs for each pair of pre- and postsynaptic cell types from three candidates: 1 thread per neuron, parallel reduction, and m threads per neuron. To choose the appropriate method, we used the value of mean convergence for each pair. If the value was smaller than 1,000, we applied the first method. If the value was larger than 5,000, then the second method was applied. Otherwise, we assigned m threads for each postsynaptic cell, so that the total number of threads for all cells of the same type is smaller than 5,192.

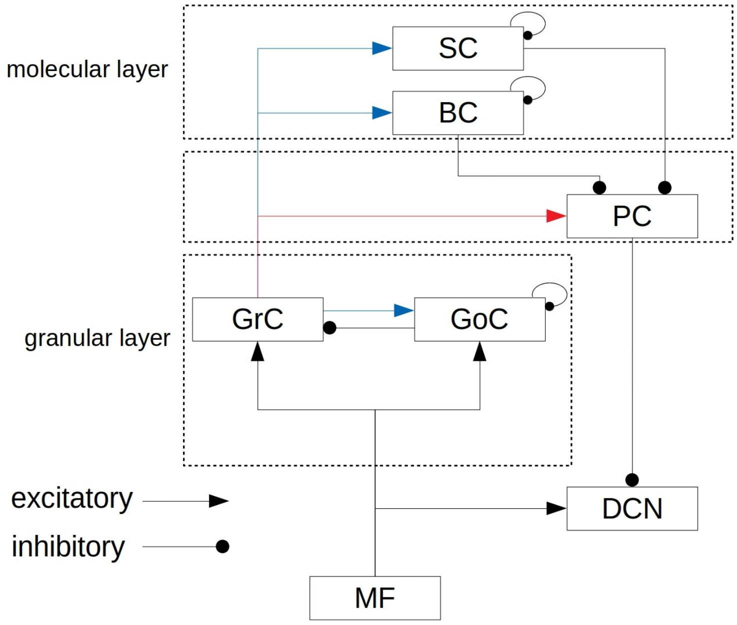

Figure S1. Parallelization methods for synaptic connection types. Colors indicate the method types: 1 thread per neuron (balck), parallel reduction (red), and m threads per neuron (blue).

2 PARAMETERS FOR THE GAIN CONTROL TASK

According to the previous study (Yamazaki and Nagao, 2012), we modified neuron-specific parameters (Table S1) and synaptic parameters (Table S2). We ignored some connections: GoC-GoC, SC-SC, BC-BC, AA-GoC, and AA-PC. Abbreviations are as in the main article.

Table S1. Neuron-specific parameters. Numbers of cells are identical to Table 1.

Type	GrC	GoC	BC	SC	PC	DCN
C_m [pF]	3.1	28	107	107	107	122.3
g_{L} [ms]	0.43	2.3	2.32	2.32	2.32	1.63
E_L [mV]	-58	-55	-68	-68	-68	-56
Δt_{ref} [ms]	1.5	2	1.6	1.6	0.8	3.7
I_e [pA]	0	0	30.0	30.0	160	500
V_{r} [mV]	-82	-72.7	-70	-70	-70	-70
V_{th} [mV]	-35	-52.0	-55	-55	-55	-38.8
τ_{exc} [ms]	1.2	1.5	8.3	8.3	8.3	10.0
τ_{inh} [ms]	7.0	-	-	-	10.0	26.6

Table S2. Synaptic parameters for each connection type.

Connection Type (exc/inh)	weight [nS]	delay [ms]
MF-GrC (exc)	0.72	4.0
GoC-GrC (inh)	0.98	2.0
PF-GoC (exc)	0.027	5.0
PF-SC (exc)	0.061	5.0
PF-BC (exc)	0.061	5.0
SC-PC (inh)	0.371	5.0
BC-PC (inh)	0.371	4.0
PF-PC (exc)	0.0042	5.0
PC-DCN (inh)	0.168	4.0
MF-DCN (exc)	0.1516	4.0

REFERENCES

Yamazaki, T. and Nagao, S. (2012). A Computational Mechanism for Unified Gain and Timing Control in the Cerebellum. *PLoS ONE* 7, e33319. doi:10.1371/journal.pone.0033319
